# Crosstalk among *Taenia crassiceps* (ORF Strain) Cysts Regulates Their Rates of Budding by Ways of Soluble and Contact Signals Exchanged between Them

**DOI:** 10.1155/2014/703693

**Published:** 2014-05-28

**Authors:** Esquivel-Velázquez Marcela, Hernández Ricardo, Larralde Carlos, Ostoa-Saloma Pedro

**Affiliations:** Instituto de Investigaciones Biomédicas, Universidad Nacional Autónoma de México, A.P. 70228, 04510 México, DF, Mexico

## Abstract

Herein we report that *in vitro* experiments with different initial parasite densities (1, 5, and 10 cysts per mL of culture medium) show that cysts at densities of 10 and 5 grow faster than those at 1, and that they release into the culture medium factors which increase the budding rates of the slower lower-density ones. Close contact among the incubated cysts also favors budding, thus suggesting the participation of surface sensors of parasite crowding. Thus, contact signals, together with the release of soluble growth factors, could endow cysts with the capacity to sense and regulate their numbers inside their habitat in relation to their population density.

## 1. Introduction

*Taenia crassiceps* is a cestode parasite which naturally affects canines and murines as definitive and intermediary hosts, respectively [1]. When* T. crassiceps* metacestodes are experimentally placed inside the peritoneal cavity of receptor mice, the cysts rapidly initiate their asexual reproduction through the daily generation of numerous visible and easily accountable buds [2]. Once parasites harbor in the peritoneal cavity of susceptible mice (i.e., Balb C/AnN), their numbers grow exponentially until reaching maximal parasite loads in the order of a couple of thousands per infected mouse, which together occupy as much volume as the mouse itself, some 3-4 months after infection, without causing apparent illness to their host.

Many host and parasite factors influence the early rates of parasite growth, such as genetic background, sex, and immune status of the host, as well as the original strain of the parasite, but rarely if ever does the intensity of infection rise above the levels mentioned.

While studying the effects of the genetic background, sex, and immunological status of mice, as well as of the time elapsed after intraperitoneal infection with* T. crassiceps* ORF cysts, we noticed that parasite loads and antibody response of individual mice varied considerably within the same strain and sex of infected mice even when the mice were infected with an equal number of apparently identical cysts collected from the same donor female mouse [3]. Discarding technical error as a comparatively negligible source of variance in murine cysticercosis experiments [4], large part of the individual host response variation in murine cysticercosis could also be attributed to the parasites inside it. This alternative source of variation was not considered before because no major genetic differences among individual cysts were expected as they derive from a single strain of parasites (ORF) and also because budding is an asexual form of parasite reproduction with reduced chances of genetic recombination as a source of diversity [5].

So, we set out to test the hypothesis that a significant source of variation lies in the reproductive capacity of each of the apparently identical cysts contained in the inoculums, each possibly having different budding capacity at the time of their inoculation, associated perhaps with their degree of individual age and differentiation. Accordingly,* in vitro *budding rates of 1, 5, or 10 cysts per mL of culture medium were registered microscopically at different days after culture, together with a visual record of their appearance, motility, and physical relation with each other. The* in vitro* experiments demonstrated that increasing parasite densities increased their rate of budding, as if the faster cysts recruited the laggard ones into faster budding [6]. Herein we report that budding rate regulatory signals interchanged between parasites are behind such recruitment.

## 2. Material and Methods

### 2.1. Parasite Collection

The cysts employed in* in vitro* experiments were collected from different single donor BalbC/AnN female mice that had been infected i.p. 2 months before or more to develop massive parasite loads [7]. The donor mice were killed by etherization in accordance with our institute's ethical procedure for experimental animals treatment (http://www.biomedicas.unam.mx/_administracion/reglamentos_formatos/archivos_pdf/reglamentoBioterio.pdf) and, immediately afterwards, their peritoneal cavities were incised to release cysts into a Petri dish containing phosphate buffered saline (PBS) and 100 *μ*g/mL penicillin/streptomycin at room temperature. Typically, a significant fraction of the harvested cysts (~10–20%) corresponded to a subpopulation of tiny (0.1–0.3 mm) nonbudding, motile, and transparent cysts, from which groups of 1, 5, or 10 cysts were selected to perform the study.

### 2.2. Parasite Cultures

Nonbudding cysts were microscopically selected and were cultured in RPMI 1640 medium at 37°C for 10 days.

### 2.3. Testing for Released Growth Factors

The culture media (1 mL) from cultures of cysts at a density of 5 or 10 cysts per mL were transferred daily, for 10 days, to wells which contained single cysts. The number of buds produced by singly cultured cysts was counted and compared with that of the control group of individually cultured cysts which received only daily fresh RPMI 1640 medium.

Transwell chambers (Costar) with a 5 *μ*m pore membrane were used to carry out a variant of the experiment of medium transfer. In this case, 1, 5, or 10 cysts were placed in the following combinations: 1/5, 5/1, 1/10, and 10/1 (upper chamber/lower chamber). The chambered cysts were cultured for 10 days and the number of buds on each of the individual cysts was microscopically counted. The medium was replaced by fresh medium on a daily basis.

### 2.4. Testing Effects of Parasite Contact upon Parasite Growth

Five or 10 nonbudding cysts were forcefully put in contact by placing them in the bottom of conical polypropylene 50 mL Corning tubes cut in their conical tip for this purpose and then glued with silicone onto the flat bottom of a well of a 6-well culture plate filled with 1 mL of RPMI 1640 medium and then cultured. In a parallel experiment, 5 or 10 cysts were individually placed inside the widest part of a 1000 *μ*L pipette tip cut and attached to the flat bottom of a well of a 6-well culture plate with silicone so that there was one parasite in each device to prevent contact between cysts. The well was then covered with culture medium so that the cysts shared the same medium without being in contact. The crowded and the individually placed collections of cysts were cultured for 10 days and the numbers of buds produced in both conditions were microscopically counted. The medium was replaced by fresh medium every third day.

### 2.5. Statistical Analysis

Student *t*-test was used for data statistical analysis.

## 3. Results

When cysts were collected from different donor mice, it became evident there was considerable variation in their rates of budding between donor mice, a variation which prompted us not to mix parasites harvested from various mice in subsequent experiments but use the cysts harvested from the same mouse in experiments designed to identify the nature of the budding rate regulatory signals.

**Figure 1:**
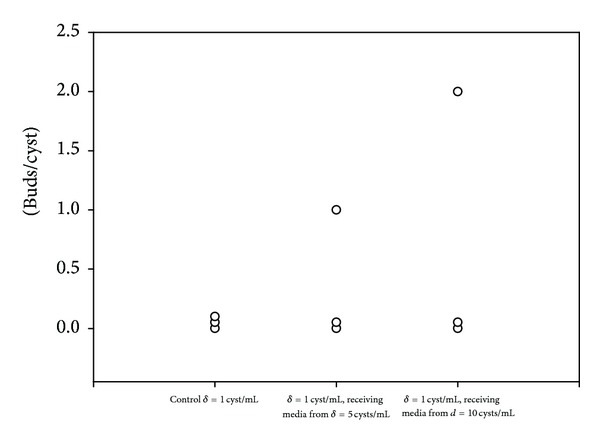
Effect of soluble factors on the budding of cysticerci. One microscopically nonbudding cysticercus/mL was cultured for over 10 days in fresh culture media; culture media from 5 cysticerci/mL or from 10 cysticerci/mL changed on a daily basis. The number of buds generated by the single cysticerci in each culture condition was counted at the 10th day. Experiments were performed by triplicate. Data represent the number of buds/cyst in each cysticercus and are plotted as individual value. Representative experiment is shown.

**Figure 2:**
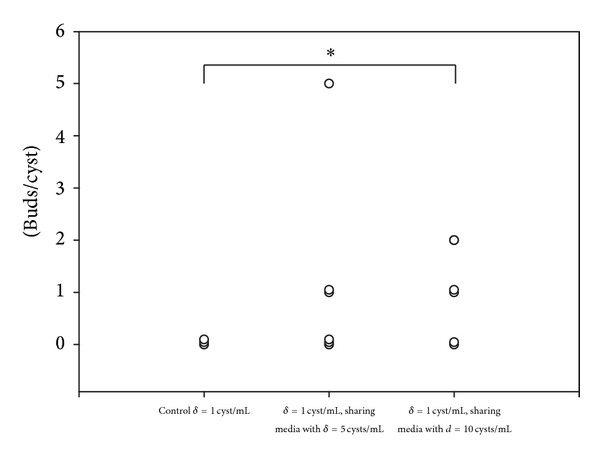
Single microscopically nonbudding cysticercus was cultured for over 10 days in a transwell system with 5 cysticerci or with 10 cysticerci on the other side of the membrane, in 1 mL of culture media. The number of buds generated by the single cysticerci in each culture condition was counted at the 10th day. Experiments were performed by triplicate. Data represent the number of buds/cyst in each cysticercus and are plotted as individual value. Representative experiment is shown. The asterisk on top of the figure represents a significant difference between the selected groups (*P* < 0.05, 2-tailed Student's *t*-test).

To ascertain if the signals from the parasite are secreted, culture medium from rapidly budding cysts (*d* = 5 or 10 cyst/mL) was transferred to cysts whose budding rate was low (density = 1/mL).  Figure 1 shows that culture medium from rapidly budding cysts (*d* = 5 or 10 cyst/mL) significantly promoted the number of buds/cyst in cysts cultured at density = 1/mL from 0.0 to 0.3 (*d* = 5) and 0.6 (*d* = 10). Also, when using the transwell system to evaluate buds/cyst when transmembranly cocultured with 5 or 10 cysts, it was found that, indeed, single cysticerci bud more effectively from 0.0 to 1.1 and 0.8 when cocultured with 5 and 10 cysts, respectively (Figure 2).

**Figure 3:**
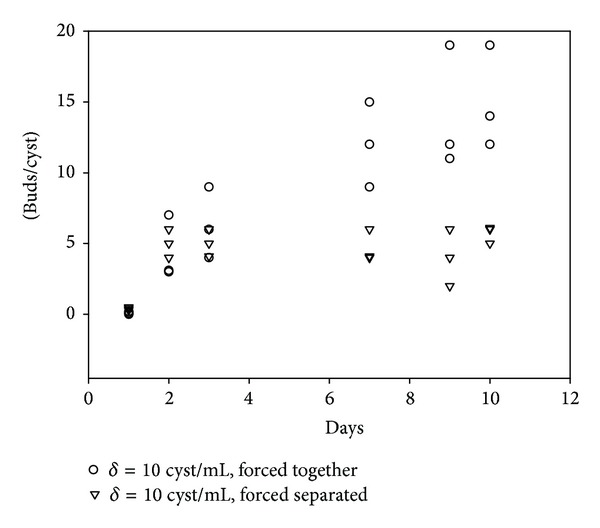
Effect of contact on the budding of cysticerci. Ten microscopically nonbudding cysticerci were cultured over 10 days in 1 mL of culture media. Cysticerci were forced to contact each other by culturing them on a conical-bottomed well or were forced to be separated. The number of buds generated in each culture condition was counted on days 1, 2, 3, 7, 9, and 10. Experiments were performed by triplicate. Data represent the number of buds/cyst in each cysticercus and are plotted as individual value. Representative experiment is shown. The asterisk on top of the figure represents a significant difference between the selected groups (*P* < 0.05, 2-tailed Student's *t*-test).

To explore if contact between the cysts was of consequence for the release of such a regulatory growth factor, we designed a simple device to force contact between the cysts and another to prevent it.  Figure 3 shows that, at the same density, the cysts bud more rapidly and efficiently when they are in contact than when they are not. Parasite contact seems to be a determining factor in a rapid budding response.

## 4. Discussion

We had previously noted that, in* in vitro* experiments, the density at which the cysts were incubated affected their rate of budding [6]. We now present evidence that there is a kind of communication between cysts in the form of a secretable molecule with an effect similar to a growth factor that enhances the rate (number of buds/cyst) of budding of cysts* in vitro*. We do not know if this factor is synthesized* de novo* by the cyst or it is a host factor absorbed by the cyst and eventually released. We favor the notion that it is synthesized by the cyst because the experiments last for several days and imply daily change of culture medium with fresh one. This argument is reinforced by the fact that the budding rate is further increased by placing the cysts in contact, probably through increased synthesis or increased release of this growth factor. Additionally, the fact that cysts contact enhances budding recalls the mechanisms like ligand-receptor interaction might be involved in the process as it happens in other systems [8]. The crowding cysts at density = 10 per se promote the highest budding efficiency which indicates that crowding is a powerful factor controlling the population of cysts even more than with hormone supplementation[6]. For that reason we assume that the phenol red in the medium has, if any, a very little effect over the budding.

We do not know the kind of signaling pathway involved in this phenomenon in* T. crassiceps*. It is known that families of genes important for signaling and development evolved before the divergence of the lineages of sponges and eumetazoan and are, therefore, in all animal lineages. [9]. The similarity of these systems between different phyla allows us to assume that the secretion of this growth factor* in vivo* can influence different host cells from organized neuroimmunoendocrinological networks [10, 11]. Likewise does the liver parasite* Opisthorchis viverrini*, which secretes mitogenic factors that can induce proliferation of host cells leading to a cholangiocarcinoma [12].

In conclusion, the* in vitro* studies presented here show that* T. crassiceps* secretes factors that affect the rate of budding of cysts and that these factors may change depending upon the host, time of infection, or contact between parasites.
